# Whole transcriptome profiling of the vernalization process in *Lilium longiflorum* (cultivar White Heaven) bulbs

**DOI:** 10.1186/s12864-015-1675-1

**Published:** 2015-07-28

**Authors:** Carlos Villacorta-Martin, Francisco F. Núñez de Cáceres González, Jorn de Haan, Kitty Huijben, Paul Passarinho, Maya Lugassi-Ben Hamo, Michele Zaccai

**Affiliations:** Genetwister Technologies B.V., P.O. Box 193, NL6700 AD Wageningen, The Netherlands; Department of Life Sciences, Ben Gurion University of the Negev, P.O. Box 653, Beersheva, 84105 Israel; Present address: Centro de Investigaciones Biológicas, Universidad Autónoma del Estado de Hidalgo, Carretera Pachuca-Tulancingo Km. 4.5, C. P. 42184 Mineral de la Reforma, Hidalgo Mexico

## Abstract

**Background:**

Vernalization is an obligatory requirement of extended exposure to low temperatures to induce flowering in certain plants. It is the most important factor affecting flowering time and quality in Easter lily (*Lilium longiflorum*). Exposing the bulbs to 4 °C gradually decreases flowering time up to 50 % compared to non-vernalized plants. We aim to understand the molecular regulation of vernalization in Easter lily, for which we characterized the global expression in lily bulb meristems after 0, 2, 5, 7 and 9 weeks of incubation at 4 °C.

**Results:**

We assembled de-novo a transcriptome which, after filtering, yielded 121,572 transcripts and 42,430 genes which hold 15,414 annotated genes, with up to 3,657 GO terms. This extensive annotation was mapped to the more general GO slim plant with a total of 94 terms. The response to cold exposure was summarized in 6 expression clusters, providing useful patterns for dissecting the dynamics of vernalization in lily. The functional annotation (GO and GO slim plant) was used to group transcripts in gene sets. Analysis of these gene sets and profiles revealed that most of the enriched functions among genes up-regulated by cold exposure were related to epigenetic processes and chromatin remodeling. Candidate vernalization genes in lily were selected based on their sequence similarity to known regulators of flowering in other species.

**Conclusions:**

We present a detailed analysis of gene expression dynamics during vernalization in *Lilium*, covering several time points and accounting for biological variation by the use of replicates. The resulting collection of transcripts and novel isoforms provides a useful resource for studying the changes occurring during vernalization at a fine level. The selected potential candidate genes can shed light on the regulation of this process.

**Electronic supplementary material:**

The online version of this article (doi:10.1186/s12864-015-1675-1) contains supplementary material, which is available to authorized users.

## Background

*Lilium longiflorum* (Easter lily) is a leading bulbous crop worldwide and is produced as cut flower, potted plant, garden plant and as dry cell bulb [1]. Like many other ornamental bulbs [2], *L. longiflorum* flowering requires cooling of the bulbs to meet the obligatory vernalization requirement of this plant species [3, 4]. *L. longiflorum*, (cultivar White Heaven) plants developing from non-vernalized bulbs grown at a constant temperature of 25 °C produced only leaves and did not flower over a period of more than 15 months (Ram *et al*., in preparation), confirming the obligatory cold requirement of this cultivar. Vernalization is also the main parameter involved in flowering time regulation in Easter lily and therefore has been the focus of a considerable amount of research related to physiological aspects of this species’ development, in order to reach flowering at specific dates [3, 5]. Typically, cold exposure of *L. longiflorum* bulbs at 2 to 10 °C quantitatively hastens flowering time while decreasing height, leaf and flower number, up to a saturation point of 6 weeks, after which additional cold exposure will not have a further effect on these parameters [3, 5–10]. In a previous study on *L. longiflorum* cultivar White Heaven [11], we found that bulb exposure to 4 °C for one week induced a decrease of about 20 % in the time from planting to floral transition and to flowering. Additional cold exposure led to a gradual decrease up to about 80 % and 55 % for floral transition and flowering, respectively, after nine weeks at 4 °C.

Despite the importance of vernalization in *Lilium* flowering, the molecular regulation of this mechanism is largely unknown in this species and other ornamental flowering bulbs. Most of the information available on molecular control of vernalization comes mainly from work performed on *Arabidopsis*, cereals and sugar beet and revealed that, while the general mechanism of vernalization is conserved among distant species, the sequence of the main regulatory genes is not [12–17].

In *Arabidopsis*, *FLOWERING LOCUS C* (*FLC*), a MADS-box gene encoding a potent repressor of flowering, is active in meristems in autumn. Flowering repression by FLC is mediated by its binding to major genes that promote flowering, such as *FLOWERING LOCUS T* and *D* (*FT* and *FD*, respectively) and *SUPPRESSOR OF OVEREXPRESSION OF CONSTANS1* (*SOC1*) [18, 19]. While FLC represses genes that induce meristems to form flowers, it relies on FRIGIDA (FRI) to elevate its autumnal expression to a level that prevents flowering [19, 20]. During winter, vernalization causes the acquisition of meristem competence to flower by repressing *FLC* expression. Once it has been repressed by vernalization, *FLC* remains off for the rest of the plant’s life cycle after the return of warm conditions, *i.e*. the repression is epigenetic in the sense that it is mitotically stable in the absence of the inducing signal (cold exposure). The mechanism of epigenetic repression of *FLC* involves histone modifications that convert *FLC* into a heterochromatin-like state. A key player in the vernalization-mediated silencing of *FLC* is *VERNALIZATION INSENSITIVE 3 (VIN3*), which is required for all *FLC* chromatin modifications associated with vernalization-mediated silencing and as a measure of the cold period [21]. Recently, it was shown that all members of the VIN3 family act together to repress *FLC* family members during vernalization [22]. In addition, the non-coding (nc) antisense transcript *COOLAIR* and the intronic long ncRNA *COLDAIR* are upregulated at different points during cold exposure and are apparently playing a role in the epigenetic regulation of *FLC* [23–25]. Altogether, this measure of gradual cold acquisition ensures that only a prolonged cold exposure (the winter season) will lead to activation of the vernalization process.

In winter cereals, which require vernalization, a system similar to that in *Arabidopsis* exists. Specifically, a flowering repressor prevents flowering prior to cold exposure and the expression of this repressor is turned off by cold. In wheat, the repressor is a zinc-finger type protein VERNALIZATION 2 (VRN2). One of the genes repressed by VRN2 is *VERNALIZATION1* (*VRN1*), which encodes a MADS-box protein that promotes flowering [26, 27]. When expressed in the leaves at high levels, VRN2 also represses *FT* [27], thereby playing a similar role as *FLC* in *Arabidopsis*. However, these two genes are unrelated and no *FLC* orthologues have been isolated from grasses [16]. In sugar beet, three genes have been found to regulate the vernalization response, *BvBTC1*, *BvFT1*, and *BvFT2* [14]. Both *BvFT1* and *BvFT2* belong to the phosphatidylethanolamine-binding protein (PEBP) family and act in an antagonistic way: overexpression of the flower repressor *BvFT1* leads to the repression of *BvFT2* (the homologue of *FT*) and delays flowering time. Furthermore, like *FLC* in *Arabidopsis*, *BvFT1* is downregulated by cold exposure. Different alleles of the *BvBTC1* locus are associated with the expression level of *BvFT1* and *BvFT2* and with the vernalization response and flowering habit of various beet genotypes [15].

High throughput sequencing can produce a wealth of information on the genes involved in a certain process. Individual genes can be annotated based on the prediction of open reading frames and by comparison with expressed sequence tag (EST) collections. For example, such technology was successfully used to analyse the transcriptome of vernalization and gibberellin responses in sugar beet, revealing connections between gene expression patterns, genotypes and treatments, as well as potential new functions of the RAV1-like AP2/B3 domain protein in the vernalization response [28]. In *Lilium*, several studies based on ESTs for genetic analyses and marker development are available [29–32]. A report about the transcriptome of Asiatic lily hybrid (cultivar Tiny Ghost) bulbs 27 days after exposure to 25 °C or 4 °C was recently published. The reported results revealed changes in the expression of a large number of genes belonging to several metabolic pathways and orthologs to vernalization genes in other plant species [33]. In that study, the length of cold exposure of the bulbs was established according to the peak in sugar content in the scales, however, the report does not specify the effect of cold exposure on flowering time, therefore, a definite association between the results and the actual vernalization process is not explicit.

The search for genes regulating vernalization in lily, which has a huge genome and no vernalization mutants available, can be narrowed with a selection of genes differentially expressed during cold exposure. In this study, we use RNA sequencing to investigate gene expression patterns in the meristems of lily bulbs at several time points during cold exposure, aiming to characterize the gradual changes occurring in the bulb during vernalization, in view of its quantitative effect on flowering.

## Methods

### Design of the experiment, plant material and RNA collection

Previous experiments conducted in our lab [11] have shown that exposure of *L. longiflorum* cultivar White Heaven bulbs to 4 °C reduces the time from planting to flowering in a quantitative manner. Therefore, several time points between 0 and 9 weeks of cold exposure were selected for the transcriptome construction in order to cover a wide range of vernalization related transcription patterns useful for later inference.

**Fig. 1:**
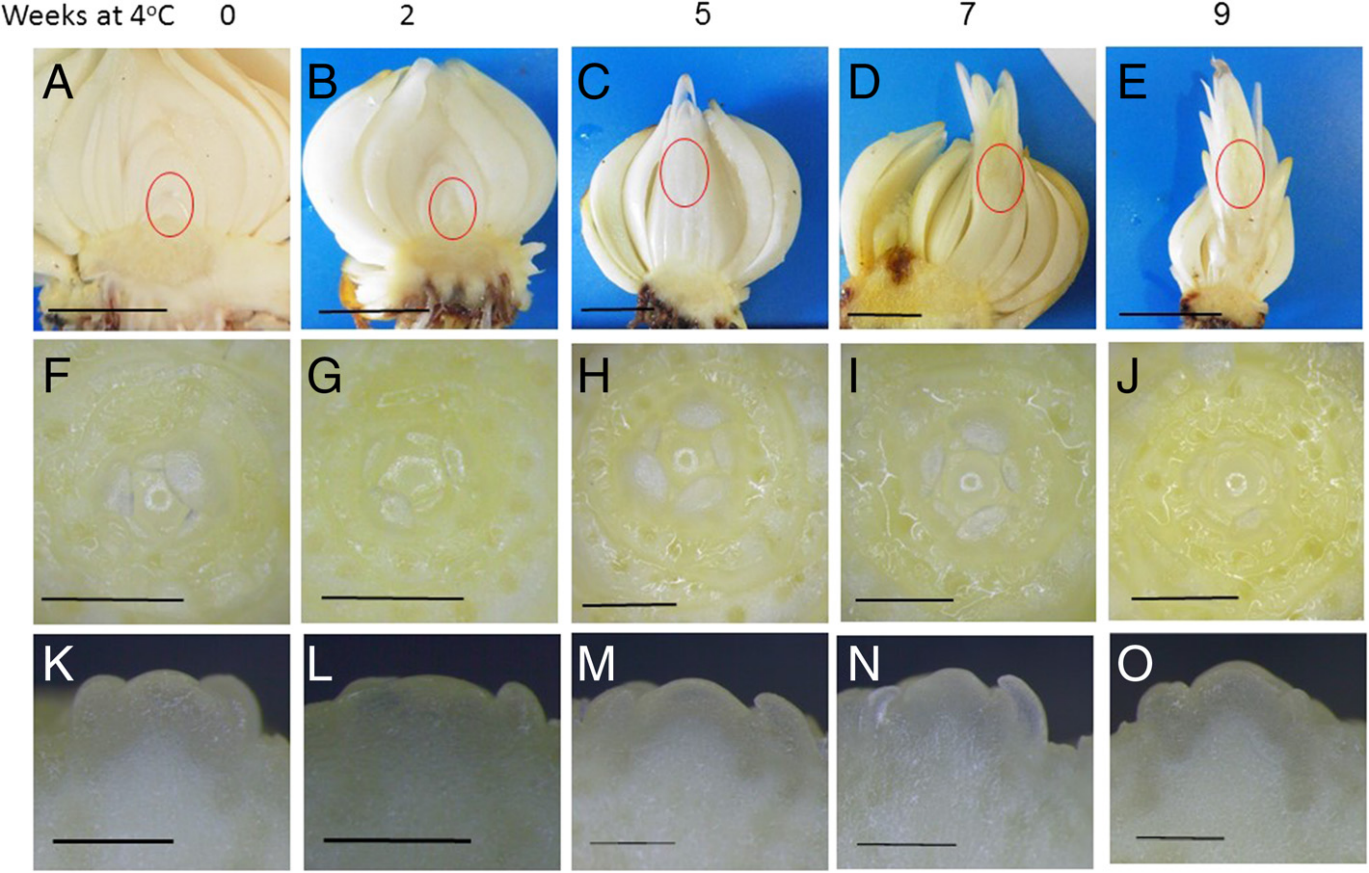
Lily bulb meristems at sampling points after 0, 2, 5, 7 or 9 weeks at 4 °C. **a**-**e**: Longitudinal section in lily bulbs. The area comprising the meristem is indicated by a red ellipse (Bar = 1 cm). Some of the outer scales have been removed. **f**-**j**: apical meristem from above, after removal of scales and leaf primordia (Bar = 1 mm). **k**-**o**: longitudinal section of the apical meristem (Bar = 0.5 mm)

*L. longiflorum* bulbs (cultivar White Heaven) were obtained from a nursery at the end of the growing season (August). Bulbs were sanitized and stored in humid standard pot medium at 25 °C (control, 0 W) or at 4 °C for 2, 5, 7 or 9 weeks (2 W, 5 W, 7 W, 9 W, respectively). At each of these time points, shoot apical meristems were excised from the bulbs, immediately frozen in liquid nitrogen and then stored at -80 °C until RNA extraction. Typically, the material used for extraction was meristem-enriched, including the meristem itself and a small portion of the stem underneath (Fig. 1a-e). Apical meristems from additional bulbs were sampled at each time point for developmental stage validation under a stereo microscope (Stemi 200 °C, Zeiss, Germany). At all points, the meristems were at the vegetative stage, as can be seen from Fig. 1f-o. Floral transition occurs after stem emergence and production of a number of leaves above ground [11]. Therefore, changes in gene expression taking place during bulb cold exposure cannot be related to a change in developmental phase of the meristem (e.g. from vegetative to reproductive), as occurring in other geophytes [2].

In order to obtain sufficient RNA for each extraction, five meristems were pooled. The number of individuals to be pooled is a compromise between the technical requirements of the library preparation protocol, the need to reduce the impact of potential outliers and the soundness of downstream statistical analysis: pooling too many individuals could lead to an underestimation of the variability of expression between replicates and thus make many false positive rejections of the null hypothesis when comparing different treatments, inflating the number of differentially expressed genes.

Two biological replicates were made for each time point. This detail of the experimental design is important, since one of the central goals of our study is to compare the expression in different stages of the vernalization process, for which we need to infer the expression variance in each condition [34]. Moreover, only using biological replication can we test whether the difference between two samples with different treatment is stronger than what we expect to see between two samples that are replicates, and therefore, whether that difference can be attributed to the cold treatment.

#### RNA-seq sample preparation and sequencing

RNA was isolated using the Aurum^TM^ Total RNA mini kit (Biorad # 732-6820) following manufacturer’s instructions. The procedure was followed by an additional DNase treatment with TURBO™ DNase (Life technologies # AM2238) to remove all genomic DNA still present after the isolation. The RNA integrity was confirmed using the 2100 Bioanalyzer (Agilent Technologies).

The samples were prepared for transcriptome sequencing using the Illumina kit (TruSeq RNA Sample Preparation Kit v2, # RS-122-2001) following the manufacturer’s recommendations. The obtained libraries were adjusted and pooled at a concentration of 20 nmol and sequencing was performed with 100-bp, paired-end reads on the HiSeq 2000 (Illumina).

### Quality control and data pre-processing

After base calling via the Illumina pipeline (HiSeq Built-In Software), a total of 528 million of paired-reads (MPR) was obtained. A stringent quality control was then applied by removing the first three bases in the 5’ ends of each read and also the bases at the 3’ ends with a quality score smaller than 20. Reads containing primer/adaptor sequences were removed as well as the ones with long mono and di-nucleotide repeats.

### De-novo transcriptome assembly

Four methods for the de-novo transcriptome assembly were tested: tIDBA [35], trans-Abyss [36], Oases [37] and Trinity [38]. The resulting assemblies were compared using a reference set of *L. longiflorum* ESTs and the criteria proposed by Martin *et al*. [39].

Considering the resulting metrics of accuracy, completeness and contiguity, Trinity was chosen to perform the final assembly because it showed the best performance for this dataset [40]. In order to obtain a transcriptome reference sequence that is common to all the data points, the reads were merged from all the different treatments before the final assembly. Since all samples were from the same lily cultivar, which is vegetatively propagated, merging different samples did not increase the complexity of the assembly. The cut-off for minimum contig length was set at 200 bp. Parameters for the assembly and further bioinformatic analyses are provided in Additional file 4.

### Mapping, summarization, filtering and exploratory analysis

Each sample was mapped separately using a Bowtie [41] wrapper bundled with Trinity. After this, the counts of mapping reads per contig and per gene were summarized. Variation between replicates and distance between samples was explored using heatmaps and principal components analysis (PCA). For this purpose we used a subset of the top 500 genes with most variable expression after variance stabilizing transformation (which renders the expression values homoscedastic in relation to the fitted expression mean). This allowed us to rule out unexpected batch effects.

In order to reduce the amount of chimeric transcripts and noise, sequences whose total read count over all conditions was below the 80^th^ percentile of read counts were filtered out. That resulted in 42,430 genes passing this filter, a number coherent with the amount of genes found in other plant species. Further filtering was applied to remove isoforms supported by less than 4 % of the reads mapping in any given gene. The purpose of independent filtering is to get rid of genes that, if tested, would have no chance of showing significant evidence of differential expression. By using a statistically independent filter (one that does not depend on the test statistic), a strong multiple-test correction (with the procedure described by Benjamini and Hochberg, [42]) was avoided and thus, statistical power was increased.

### Differential expression analysis

Differential expression between each pair of conditions was analyzed following the methods as implemented in DESeq v1.10.1 [34]. Robust estimates of the variability in the expression of each gene were calculated doing an average of its variability over each condition (weighted by the number of samples for each combination of factors, which is the same for all conditions). For a confident dispersion estimate, a conservative approach was used: estimates of high dispersion genes (those above the fitted regression line) were accepted but estimates of low dispersion genes were pulled up to the fitted line [34]. We used a corrected [42] *p*-value of 0.05 as threshold to call differentially expressed genes.

### Gene clustering

After analyzing differential expression, we extracted genes beyond a threshold of a 4 fold-change in expression between any pair of time points and clustered them according to their patterns of differential expression across time. This was made using both the standard K-means clustering algorithm and pre-computed models for time series expression data in STEM [43]. In particular, this last method first defines a set of distinct and representative models of gene expression, all of whom use a value of zero as a starting point. The number of real gene profiles assigned to each model is then computed. The number of genes expected to be assigned to a profile is estimated by randomly permuting the original time point values, renormalizing the gene’s expression values and then assigning genes to their most closely matching model profiles, repeating this for a large number of permutations. The average number of genes assigned to a model profile over all permutations is the expected number of genes for that model. This provides a framework to test the statistical significance of profile clusters. The biological significance of the model or cluster was investigated by means of GO [44] enrichment analysis, as explained in the gene set analysis section. For this clustering, those profiles that did not show a minimum absolute expression change of 4 log-fold (based on the difference between the minimum and maximum) were filtered out.

### Functional annotation

Function prediction was done in three steps: first inferring ORFs with Transdecoder (bundled with Trinity, [45]), second, calculating sequence similarity to a protein sequence database (UniProtKB) and third, to a protein profile database [46].

Computational approaches to annotation is not straightforward due to ambiguities in the model sequence-structure-function. Therefore, it is important to back inferences in different sources that should be integrated according to the semantics and strength of the evidence for each annotation source. For this integration purpose, we used Argot2 web-service [47]. We provided as an input the results of sequence-based methods (Blast to a relevant subset of UniprotKB: plant sequences with a GO annotation) and functional domains assignments (HMMER3 [48] to PfamA profiles [46]).

For those genes whose ORFs could not be inferred or could not be annotated, an additional similarity search (DNA to protein database) was done using Blastx and mapping the GO terms to the Blast results with Blast2GO [49]. Once all the relevant GO annotations were obtained, these were mapped to GOslim Plants to achieve a reduced representation of GO terms.

### Gene set analysis

The purpose of gene set analysis (GSA) [50, 51] is to shift the focus from single genes to sets of related genes. GSA was performed using two very different approaches: first, over gene profiles (clusters of genes with similar time-series expression patterns) and second, over pairwise comparisons of differential expression performed between different vernalization treatment time-points.

For the first approach, GSA was performed using time-series profiles with STEM [43]. In this way, the cluster significance was determined first (checking whether the number of genes in each profile is higher than expected by chance alone), and afterwards their biological significance using an enrichment calculation based on their GO annotation and on the expected number of genes annotated with that specific GO given that particular cluster size. This calculation involves a comparison to a base set of genes (i.e. background or complement used as a reference) which in this case was comprised by all the genes in the dataset. Gene sets were based on the functions and pathways represented by the initial GO annotations (each of the 3657 ontology terms defines a gene set) and also on the transposons and other repetitive elements annotated with RepeatMasker [52].

In the second approach, GSA was performed with the package for R/Bioconductor GSVA 1.4.4 [53]. This uses as input a gene expression matrix in the form of read counts and a database of gene sets, which were defined over the whole transcriptome, using GO slim plants (a summarized version of the 3657 GO annotations that results in 94 more general terms) and a subset of the transcriptome corresponding to genes annotated as transposons, using RepeatMasker [52] for that purpose (as in the previous method). This package implements a non-parametric unsupervised method for assessing gene set enrichment. It calculates gene expression level statistic for each sample and then it orders them by rank. Next, a Kolmogorov-Smirnov-like rank statistic is calculated which is subsequently used to obtain the resulting GSVA enrichment scores.

### Orthologue vernalization gene finding

In order to find genes related to vernalization, several approaches were followed. First we used an “omics” approach, already described in the functional annotation sections. We selected genes whose inferred ORFs were annotated with terms related to vernalization. From these sequences we selected those whose domains matched the ones present in genes known to be involved in vernalization from other species (Arabidopsis [54], rice [55] and wheat [56]). Then, the protein sequence of each of the lily candidate genes was blasted [57] against the UniProtKB database. Top hits were then filtered based on the highest percentage of hit coverage combined the highest percentage of sequence similarity. Expression profile from the top hits was used to compare it against the expression of their putative orthologues and used it as another filtering criterion. Additionally we blasted protein sequences of known genes from other species, involved in vernalization, against the transcriptome data using BioEdit software [58] Top hits were then selected and filtered as described previously.

## Results

### Transcriptome statistics

The initial Trinity assembly yielded a total of 329,599 transcripts belonging to 212,304 components (potential genes). By filtering out genes with lower count numbers and removing isoforms that represent just a small fraction of a gene’s total counts (smaller than 4 %) we reduced the total number to 121,572 transcripts and 42,430 genes. The total size of the unfiltered transcriptome was 263,039,169 bp. The N50 was 1,443 bp. The longest contig was 16,512 bp and the shortest, 201 bp. G + C content was 42.3 %.

### Differential expression analysis

**Fig. 2:**
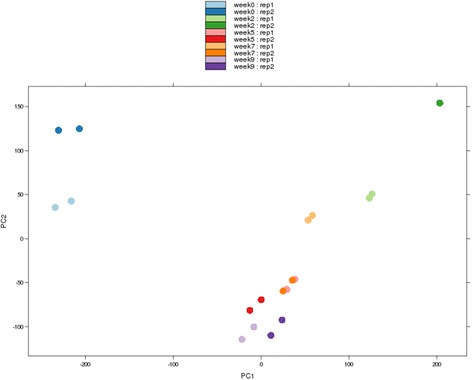
PCA of technical and biological replicates for each time point. Each hue corresponds to a different time-point. Each time-point has two biological replicates which are represented in different tints. Technical replicates are represented with exactly the same hue and tint. Technical noise is small. Most variation occurs between time-points 0 W and 2 W. No unexpected batch effects take place, other than the controlled experimental factors

Our exploratory analysis (Fig. 2) showed that there are no unexpected batch effects and that replicates tend to cluster with each other (particularly so in the case of technical replicates). A critical step in the analysis of gene expression data is the detection of differentially expressed genes. As shown in Table 1, most changes in expression took place during the first 2 weeks of cold exposure (9,872 genes, time-points 0-2). During the next three weeks, the number of differentially expressed genes was lower (1,938 genes, time-points 2-5) and expression appeared almost stable in the following measured periods (19 genes, time-points 5-7 and 81 genes, time-points 7-9).

**Table 1:** Final numbers of differentially expressed genes and isoforms differentially expressed (DE). Between pairs of vernalization treatments (0 to 9 weeks at 4 °C) using the most conservative parameters

Type	*n*	0-2	0-5	0-7	0-9	2-5	2-7	2-9	5-7	5-9	7-9
Genes	42,430	9872	7574	6651	7864	1938	427	3584	19	444	81
Isoforms	121,572	7576	6244	5377	7409	643	491	1196	134	477	271

**Fig. 3:**
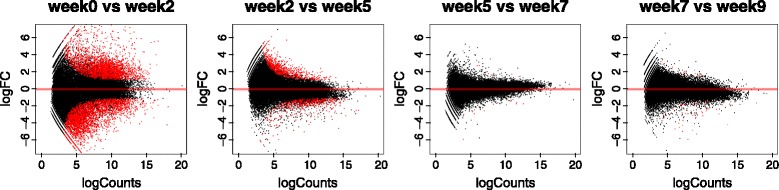
MA plots of differentially expressed genes in a 2 or 3 week window. Each dot represents a gene. The x-axis represents the logarithm of read counts in that gene. The y-axis is the logarithm with base 2 of the fold change from one condition to the other (logFC = 2 implies a 4 fold-change in expression). Red dots represent differentially expressed genes (adj. *p*-val < 0.05)

The number of genes significantly upregulated appears similar to the number of downregulated genes (Fig. 3, red dots). Notice as well that the fold changes in expression were higher during the first weeks of cold treatment.

### Clustering and time-series analysis

Significant temporal expression profiles and the genes associated with these profiles were identified by clustering and visualization of the time series expression data. We integrated gene ontology data to perform enrichment analyses for sets of genes having the same temporal expression pattern.

**Fig. 4:**
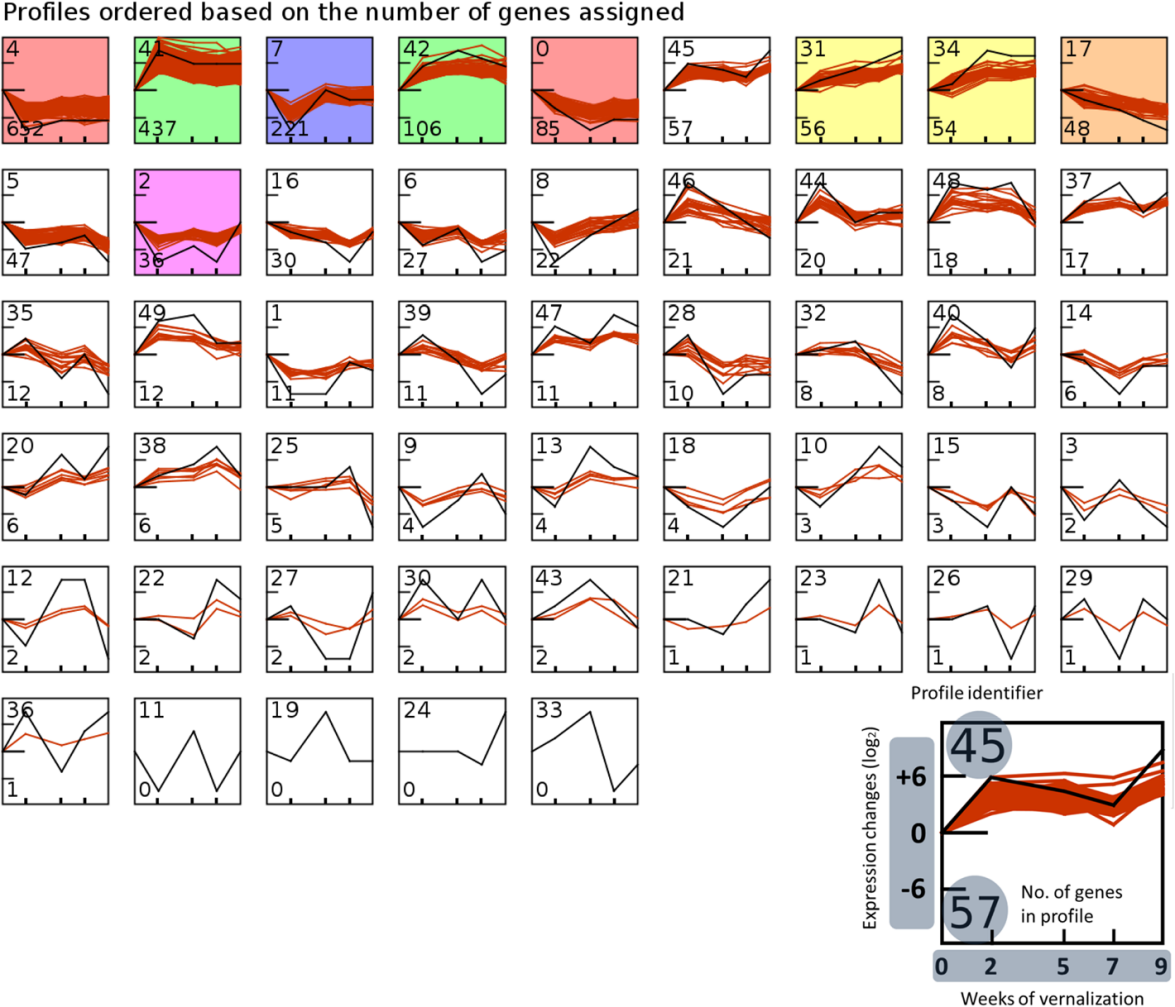
Expression profiles during cold exposure (from 0 to 9 weeks) ordered by the number of genes assigned. As indicated on the graphic legend at the bottom right, the number on the upper left of each profile is the profile ID. The number in the bottom left indicates the number of genes assigned to the profile during clustering. Profiles with colored background are significant in terms of the number genes assigned to them in comparison to random permutations. Profiles colored with the same shade belong to the same cluster (correlation > 0.7)

From the 42,430 total genes we selected for time-series analysis only those with a minimum absolute expression change between time-points of 4-fold resulting in a total of 2,095 genes. A set of 50 pre-computed expression patterns [43] was used; each of them was called a model profile. Some of these models were very similar to each other; thus if this similarity was beyond the minimum correlation coefficient of 0.7 then these model profiles were grouped under the same cluster (profiles represented with the same color, Fig. 4). This distinction between profiles and clusters applies only to the STEM-specific algorithm. Each square in Fig. 4 represents a profile and each group of profiles with the same color a cluster (i.e. 50 profiles, 6 clusters). Profiles that had a significant amount of genes (more than expected by chance alone) are colored. Using more pre-computed profiles yielded similar results (data not shown). The pre-computed model profiles were generated using a value of 2 for the maximum unit change parameter (c). Additional tests with c = 1 and c = 3 returned similar results (data not shown). From the 50 model profiles, nine profiles in six clusters were identified as significant. A minimum correlation of 0.7 was used as a threshold to group profiles in the same cluster, where the value of 0.7 was obtained by considering the average distance between the two data replicates. Of the six clusters of profiles, three contained two profiles and three were single profiles. four of the nine significant model profiles had significantly enriched GO categories (based on a hypergeometric test), two of these profiles were in an upregulated cluster (#41 and #42, Fig. 4) and the other two were in a downregulated one (#4 and #0, Fig. 4). We noted that the transcriptome still contains a large number of unannotated genes, which could explain why other profiles were not significantly enriched for GO categories. Table 2 describes the functions enriched for the genes in the profiles mentioned above.

**Table 2:** Overrepresented gene sets in the upregulated and downregulated expression time-series clusters. Genes are categorized in sets according to their Gene Ontology. Column “Observed” is the number of genes from each category or set assigned to the cluster. ”Expected” is the number of genes that was calculated in the permutation simulation based on the total size of each category and the uniqueness of the profile. The results are order according to the Fold change. Only significant results are shown (Bonferroni corrected *p*-value < 0.05)

GO enrichment for Cluster 1 upregulated (Profiles 41 and 42)					
Category ID	Category Name	Observed	Expected	Fold	Corrected *p*-value
GO:0006270	DNA replication initiation	5	0.1	35.5	<0.001
GO:0044815	DNA packaging complex	13	0.5	27.5	<0.001
GO:0000786	nucleosome	11	0.4	26	<0.001
GO:1990104	DNA bending complex	11	0.4	26	<0.001
GO:0034728	nucleosome organization	11	0.5	22	<0.001
GO:0006334	nucleosome assembly	11	0.5	22	<0.001
GO:0065004	protein-DNA complex assembly	12	0.6	20.8	<0.001
GO:0071824	protein-DNA complex subunit organization	12	0.6	20.8	<0.001
GO:0031497	chromatin assembly	12	0.6	20.8	<0.001
GO:0032993	protein-DNA complex	18	0.9	19.3	<0.001
GO:0006333	chromatin assembly or disassembly	12	0.6	18.8	<0.001
GO:0006323	DNA packaging	14	0.8	18.2	<0.001
GO:0006261	DNA-dependent DNA replication	7	0.4	17.1	<0.001
GO:0000785	chromatin	11	0.7	16.2	<0.001
GO:0044427	chromosomal part	22	1.6	13.8	<0.001
GO:0071103	DNA conformation change	16	1.2	13.6	<0.001
GO:0000079	regulation of cyclin-dependent protein serine/threonine kinase activity	5	0.4	11.5	0.022
GO:0071900	regulation of protein serine/threonine kinase activity	5	0.5	10.9	0.03
GO:0046982	protein heterodimerization activity	9	0.8	10.8	<0.001
GO:0019901	protein kinase binding	5	0.5	10	0.038
GO:0019900	kinase binding	5	0.5	10	0.038
GO:0043549	regulation of kinase activity	5	0.5	9.3	0.05
GO:0045859	regulation of protein kinase activity	5	0.5	9.3	0.05
GO:0005694	chromosome	24	2.7	9	<0.001
GO:0051726	regulation of cell cycle	10	1.1	9	<0.001
GO:0008017	microtubule binding	6	0.8	7.6	0.042
GO:0034622	cellular macromolecular complex assembly	16	2.4	6.7	<0.001
GO:0006461	protein complex assembly	15	2.4	6.3	<0.001
GO:0070271	protein complex biogenesis	15	2.4	6.3	<0.001
GO:1903047	mitotic cell cycle process	7	1.1	6.2	0.042
GO:0006325	chromatin organization	18	3	6.1	<0.001
GO:0065003	macromolecular complex assembly	16	2.6	6	<0.001
GO:0007049	cell cycle	21	3.8	5.5	<0.001
GO:0051276	chromosome organization	21	3.8	5.5	<0.001
GO:0071822	protein complex subunit organization	15	2.9	5.3	<0.001
GO:0043933	macromolecular complex subunit organization	16	3.1	5.1	<0.001
GO:0022402	cell cycle process	14	2.8	5	<0.001
GO:0006281	DNA repair	13	2.6	4.9	<0.001
GO:0022607	cellular component assembly	17	3.5	4.8	<0.001
GO:0005874	microtubule	9	2	4.5	0.048
GO:0006974	cellular response to DNA damage stimulus	13	2.9	4.4	<0.001
GO:0051301	cell division	11	2.6	4.2	0.022
GO:0006260	DNA replication	22	5.8	3.8	<0.001
GO:0044085	cellular component biogenesis	19	5.8	3.3	<0.001
GO:0006259	DNA metabolic process	47	15.3	3.1	<0.001
GO:0006996	organelle organization	27	9.3	2.9	<0.001
GO:0043228	non-membrane-bounded organelle	41	14.4	2.8	<0.001
GO:0043232	intracellular non-membrane-bounded organelle	41	14.4	2.8	<0.001
GO:0043234	protein complex	43	16.5	2.6	<0.001
GO:0003677	DNA binding	44	18.6	2.4	<0.001
GO:0016043	cellular component organization	34	14.5	2.3	<0.001
GO:0071840	cellular component organization or biogenesis	35	16.3	2.2	0.006
GO:0032991	macromolecular complex	46	22.7	2	<0.001
GO:0005634	nucleus	71	40.3	1.8	<0.001
GO:0005488	binding	138	101.6	1.4	0.016
GO:0044699	single-organism process	107	76.2	1.4	0.044
GO enrichment for Cluster 0 downregulated (Profiles 0 and 4)					
Category ID	Category Name	Observed	Expected	Fold	Corrected *p*-value
GO:0006278	RNA-dependent DNA replication	20	5.5	3.7	0.002
GO:0003964	RNA-directed DNA polymerase activity	15	4.4	3.4	0.03
GO:0015074	DNA integration	24	9.4	2.5	0.028

While other profiles (like the #38, Fig. 4) showed patterns of expression matching our expectations of vernalization as a gradual cumulative process, the number of genes assigned to them in our clustering algorithm was not significant (a random pattern could have similar numbers of genes assigned just by chance) and was therefore not further explored at this stage.

In profile #41 (Fig. 4), most of the significantly enriched GO terms (corrected *p*-val < 0.05) (significant and upregulated, see Fig. 4) were related to epigenetic processes and chromatin remodeling (Table 2). From these profiles, the expression of several genes was validated by qPCR. In general, there were good correlations between the results obtained by RNA-seq and qPCR (Additional file 1: Figure S1).

### Annotation statistics and functional enrichment

From the final 121,572 transcripts, a total of 82,219 ORFs were inferred (of which 67,658 ORFs had an annotation). These ORFs correspond to 15,414 genes, which were annotated with up to 3,657 GO terms.

An inherent problem of using GO annotations to define gene sets, is the strongly unbalanced sizes of the resulting groups (some GO terms apply to thousands of genes, while other, more specific functions, are represented by fewer than 10 genes). This disparity is inconvenient for sound statistical analysis (see gene set analysis).

In order to further facilitate the description of the functions in the transcriptome and decrease the effect of expression heterogeneity in the smaller sets, we translated the previous GO annotations into GO slim plant ontologies. GO slims are cut-down versions of the GO ontologies containing a subset of the terms in the whole GO. They give a broad overview of the ontology content without the detail of the specific fine grained terms. This turns out particularly useful as a summary of the results of GO annotation.

Instead of creating a custom GO slim vocabulary or a generic “all-species” one, we used an intermediate approach: a standard GO slim plant vocabulary developed by The *Arabidopsis* Information Resource (TAIR) [54] in order to facilitate comparisons with other plant species in future studies. With this new ontology, the number of functions was summarized in 94 annotations (Additional file 2: Figure S2), which could be further tested for overrepresentation using gene set analysis.

### Gene set analysis

Gene set enrichment analysis is a way of abstracting the differential expression analysis from the level of gene expression profiles into a pathway or functional level. It provides a reduction dimensionality of the sample (testing a few hundred gene sets, rather than many thousand individual genes), as well as greater biological interpretability.

Specifically, with GSVA (Gene Set Variation Analysis) [53] we calculated sample-wise gene set enrichment scores as a function of genes inside and outside the gene set, analogously to a competitive gene set test (see Methods). Moreover, we estimated the variation of gene set enrichment over the samples independently of any class label. Conceptually, this is equivalent to a change in coordinate systems for gene expression data, from genes to gene sets.

Our first enrichment analysis (see Clustering and time-series analysis results above) was done for each of the time-course clusters using the complete GO annotation, and only 2 clusters (those with profiles 41/42 and with profiles 4/0) had functions significantly overrepresented on them and are therefore shown in detail in Table 2. Other clusters might simply not provide enough power for the hypergeometric enrichment test to detect any effect due to the smaller number of genes included or to the small number of genes annotated with each term.

This period between a non-vernalized and vernalized state, which is critical for the plant’s ability to flower in view of its obligatory requirement for cold, was characterized by major changes in gene expression (Fig. 3), which therefore might be related, among other processes, to the flowering mechanism.

**Fig. 5:**
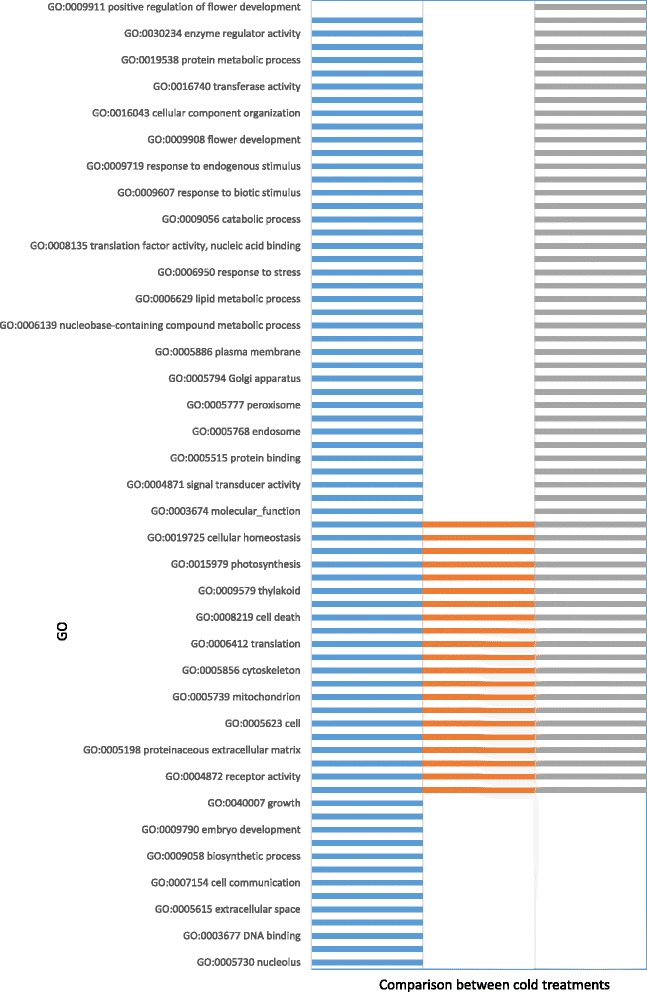
Comparisons of differentially expressed functions between non-vernalized bulbs and bulbs vernalized for 2, 5 or 7 weeks. The ID and description of the GO are presented in the y axis. Comparisons between treatments: 0-2 (blue), 0-5 (orange), 0-7 (gray) are presented in the x axis. Comparison 0-9 was also made but not shown because no significant results were obtained. All the significant results for GO sets correspond to upregulated functions (none of them was found to be downregulated)

**Fig. 6:**
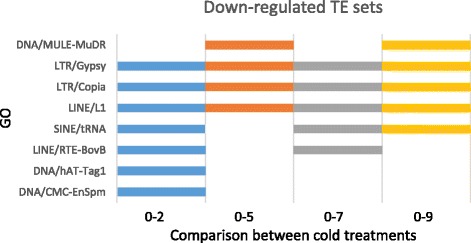
Comparisons of differentially expressed transposable elements (TEs) between non-vernalized bulbs and bulbs vernalized for 2, 5, 7 or 9 weeks. The ID and description of the TE annotations are presented in the y axis. Comparisons between treatments: 0-2 (blue), 0-5 (orange), 0-7 (gray), 0-9 (yellow) are presented in the x axis. All the significant results for TE sets correspond to downregulated functions (none of them was found to be upregulated)

**Fig. 7:**
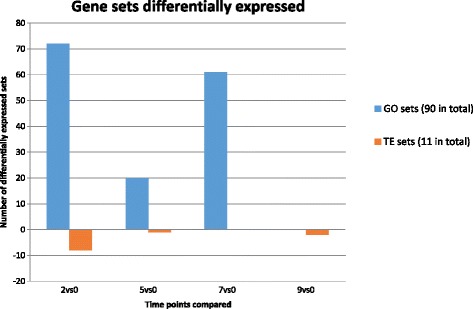
Number of GO and TE gene sets differentially expressed. Blue bars represent gene sets defined by their Gene Ontology (GO) while orange bars denote sets of genes annotated as Transposable Elements (TE). The y-axis indicates, for each of the comparisons, how many gene sets are upregulated (positive values) and downregulated (negative values)

In contrast to the time-series approach, where we used the fine-grained GO annotation of 3657 sets (and therefore, fewer genes in each set), Figs. 5 and 6 represent the results of functional enrichment using a different approach, where the compared groups correspond to different time points rather than different clusters. This was done using gene sets defined by the GO slim plant annotation, a more general classification that resulted in less sets (94) with higher number of genes in each one. Most of the gene sets with significant changes in expression were detected during the first 2 weeks of vernalization. Figure 7 summarizes the number of up and downregulated sets for each pairwise comparison, both for GO slim and transposable elements.

### Ortholog vernalization genes finding

Two approaches were subsequently followed to identify putative orthologues as described in the Methods section. After screening theses sequences we identified 12 potential candidate genes with high sequence similarity to known genes in other species known to be involved in the vernalization pathway. Alignments of the lily potential candidates with genes from other species are presented in Additional file 3: Figure S3. Some of them are key players in this biological process including *VIN3*, *SOC1*, *VRN2* and *FT* from *Arabidopsis* and others, such as *VRN A1* /*VRN1* from wheat and barley respectively. Table 3 shows the known genes from other species and their comparison with the identified putative ortholog in lily.

**Table 3:** Lily putative orthologues of vernalization-related genes and their expression in bulb meristems during cold exposure. Gene expression during cold exposure is schematically represented based on the log-fold change from week 0 to 9 at 4 °C

Gene product/Species	Putative ortholog in lily	Protein identity (%)	Conserved domains	Flowering Enhancer/Repressor	Expression profile in lily meristem during cold exposure^a^	Reference
FD / *Arabidopsis*	Comp189117-c0	72	bZIP	Enhancer	*	[71]
FT / *Arabidopsis*	Comp184710-c4	68	PEBP	Enhancer	*	[72]
SOC1 / *Arabidopsis*	Comp189264-c0	63	MADS-box, K-Box	Enhancer	*	[73]
VIP4 / *Arabidopsis*	Comp178334-c0	53		Enhancer		[74]
VRN A1, VRN1 / Wheat, Barley	Comp176908_c0	59	MADS-Box, K-Box	Enhancer	*	[75]
VRN B3, VRN H3 / Wheat, Barley	Comp178222_c0	70	PEBP	Enhancer	*	[26]
VRN2 / *Arabidopsis*	Comp184285-c0	44	Polycomb	Enhancer		[76]
EMF2 / *Arabidopsis*	Comp184487-c0	53	Polycomb	Enhancer		[77]
VIN3 / *Arabidopsis*	Comp174259-c0	41	PHD-type Zinc finger	Enhancer	*	[21]
VIP6 (ELF8) / *Arabidopsis*	Comp120922-c0	65	TPR	Enhancer	*	[78]
SUF4 / *Arabidopsis*	Comp183048-c0	53	C2H2-type Zinc finger	Repressor		[79]
SVP / *Arabidopsis*	Comp176786-c0	62	MADS-Box, K-Box	Repressor		[80]

^a^Expression of at least one point during cold exposure was significantly different (*p*-adj < 0.05) than the control (0 W)

## Discussion

### Importance of the assembled transcriptome as reference for further research

In bulbous species, large genome sizes (36 Gbp for lily) make full genome sequencing expensive and error-prone [30]. Transcriptome sequencing provides a proxy for high throughput comparisons of the exomes of crops with large genomes. In this way, ESTs of lily and tulip cultivars have been assembled using 454 pyro-sequencing, essentially for marker development [31]. The transcriptome produced in this study provides additional molecular data for lily and a reliable quantitative profile of genes differentially expressed during cold exposure, enabling the molecular analysis of the vernalization response at a global scale.

While the physiological response of the plant to cold exposure has been well characterized in the literature [3, 5–10, 59] and by our own lab on this specific cultivar [11], the transcriptome analysis provides deeper insights into the molecular regulation of this vernalization response in a major flowering species. It will also enable the isolation of genes regulating flowering, which could be used to reduce vernalization requirements and to develop molecular markers for optimal bulb cold treatment, aiming at year-round production, high flower yield and quality, and reduced economic and environmental costs. In addition, it can serve to address fundamental questions regarding the conservation of the vernalization response among higher plants.

### The emergence of systemic approaches for researching vernalization

From the methodological point of view, there is notable interest in importing the knowledge of vernalization acquired in model-species like *Arabidopsis* and wheat into other commercially relevant crops. This can be done using a singular, targeted approach -from genes to systems, or a more comprehensive, integrative approach -from systems to genes [60].

In our approach to tackle the vernalization process in *Lilium*, we have focused in the latter: the functional genomics of vernalization. To our knowledge, this is the first time in which vernalization of a bulb species is studied as a true dynamic process (including several time points), with a comprehensive level for comparative genomics (sequencing of more than 500 million read pairs yielding 121,572 isoforms) and a realistic model of expression variation (using biological replicates). With the assembly of a comprehensive de-novo transcriptome we provide the ground to develop and test hypotheses related to the dynamics of vernalization in a poorly studied, yet economically important group of plants.

During the annotation we identified novel isoforms for many potential orthologues of vernalization genes. A previous study measured expression of Asiatic lily bulbs before and after vernalization (27 days at 4 °C) using RNA-seq [33]. Both studies show that vernalization induces many cellular, biological and metabolic changes and similar genes, such as the *SOC1* homologue, are found upregulated after cold treatment in the bulb meristem hinting that this gene, known to be involved in flowering, might play a role in the vernalization pathway.

A recent report by Huang *et al*., [33] attempted to understand the process of vernalization using what they refer to as “dynamic transcriptomes”. Their expression study involved a comparison between two samples taken before and after vernalization takes place but not during the vernalization process itself. Moreover, that comparison was done without considering biological variation in the expression. The approach followed in our research was to monitor the changes in expression throughout the whole process of vernalization. It is important to mention that the need of biological replication (sequencing different cDNA libraries extracted from different individuals) for assessing the effect of a given treatment in gene expression is critical and has been addressed before [61]. The lack of this biological replication might be an explanation for the striking number of 68116 genes “differentially expressed” [33] found in the previously mentioned report by Huang *et al*., [33].

By using multiple time-points and biological replication in our research, we can say that the present work is the first dynamic study of the effect of vernalization in gene expression of lily bulbs.

The differential expression analysis for multiple time points during cold exposure allowed us to point at all the individual genes affected by vernalization at each stage. This information is critical for prioritization of downstream analysis and validation. On the other hand, gene set analysis allowed us to gain insight into the functions being influenced during vernalization and also the global effect that it has over the transcribed fraction of the transposable elements in the genome. Gene set analysis provides a scalable way to look at the changes that take place during vernalization at a high level.

The clustering performed for the time-series profiles also shows a general view on the number of genes that are up and downregulated, their functions and the patterns across time. None of these questions could be addressed adequately using an approach focused solely on individual genes. Additionally, unlike microarray studies, the use of high throughput sequencing technologies gave us margin to discover and measure new isoforms that would otherwise be unaccounted in the probes of array platforms.

### Functional enrichment during cold exposure

The difference in the number of enriched functions that were upregulated versus downregulated by cold was strikingly high (see results of gene set analysis). In agreement with experiments in wheat and *Arabidopsis*, which had previously shown that vernalization is an epigenetic phenomenon [62], Table 2 illustrates empirically that the clusters found during bulb vernalization are consistent with theoretical expectations: the vast majority of enriched genes were annotated to functions related to chromatin and chromosome modifications and to functions related to cell division. Indeed the “memory of the cold” in the vernalization process is closely linked with cell division [63]. Moreover, cell division is a pre-requisite for the integration and the maintenance of the vernalization signal [64, 65]. Therefore, upregulation of cell division-related genes during *Lilium* bulb vernalization is to be expected and is observed here.

Chromatin remodeling is an important regulatory mechanism of gene expression, well conserved among eukaryotes [66]. The series of epigenetic events taking place during cold accumulation by the plant seem to be a common mechanism in flowering plants responding to vernalization [67]. Indeed, chromatin modifications are tightly associated with the vernalization pathway in the context of the epigenetic repression of floral inhibitors. In the case of *Arabidopsis*, repression of the major floral repressor *FLC* by vernalization shows a pattern of epigenetic silencing comprising downregulation during cold exposure and continuation of silencing during the warm period following cold [62]. This mechanism involves the action of chromatin-remodeling complexes, which are responsible for histone methylation at the *FLC* locus [22]. In cereals, the vernalization response also involves epigenetic regulation. However, this regulation is not targeted to a floral repressor, but rather to the flowering enhancer *VRN1*, whose expression is silenced by histone methylations before cold exposure [68, 69]. In view of the conservation of cold-related epigenetic regulation of flowering among such distant species as *Arabidopsis* and cereals, it is probable that such a regulation exists in *Lilium* as well. The enrichment of chromatin related functions observed in the lily transcriptome during cold exposure could also be related to the silencing of transposable elements [66], whose expression decreased during cold exposure, as shown in the gene set analysis. Chromatin-related gene enrichment could also be attributed to the plant’s reponse to cold as an abiotic stress, as was observed for the ATP-dependent chromatin remodeling factors Snf2, in rice [70].

### Ortholog and candidate vernalization genes

One of our goals was to identify candidate genes that play a role in lily vernalization. Based on their expression profile and/or sequence similarity to proteins involved in vernalization in other species, we identified 12 genes. These genes were classified then into enhancers or repressors (expected to be upregulated and downregulated respectively after cold induction) based on their reported function in other species. The expression profile of some of the candidate genes in lily did not match the expression reported in other species, suggesting that they may have a different role in the pathway. These results are similar to findings in beet, in which two paralogs of the *FT* gene (in *Arabidopsis*) have antagonistic functions. *BvFT2* is functionally conserved with *FT* but *BvFT1* represses flowering and its downregulation is essential for vernalization response in this species [13]. In addition these data strongly suggests that, while the general mechanism of vernalization is relatively conserved among these distant species (a floral repressor is downregulated), the genes that mediate this process are not homologs and some of these components have resulted from convergent evolution [10–16]. For example, in wheat, although *VRN2* is considered to have the same function as *FLC* as a floral repressor, it is evolutionarily unrelated, and so far there are not known orthologues of *FLC* in monocots [15]. New functions for known genes are also hypothesized and will be subsequently validated.

In the differential expression analysis a relatively high number of genes were found to have significant changes during cold induction, which may only hint that these genes are candidates of regulators of vernalization in lily. Thus, a more thorough analysis of these data is needed. Additional experiments, including expression analysis of these and other genes in meristems, scales and leaves of lily plants grown under different environmental conditions are currently being performed, in order to further investigate the involvement of the genes in the vernalization pathway and in plant development. Furthermore, functional analysis of the selected candidate genes in lily and in *Arabidopsis*, also under way, will provide more information on how and when they are involved in the vernalization response. This will serve as a starting point to elucidate the vernalization pathway in this species and to have a detailed understanding of the molecular changes occurring in the bulb under cold induction.

## Conclusions

In this study, we presented a detailed analysis of gene expression dynamics, covering a series of time points during bulb cold exposure in *Lilium longiflorum*, a leading ornamental crop. The resulting collection of transcripts and their novel isoforms provides a valuable data base for the exploration of fine molecular changes occurring during vernalization and the selected potential candidate genes can lead to the elucidation of the molecular regulation of vernalization in lily.

Overall, this study constitutes an important contribution to our current understanding of the molecular regulation and evolution of the vernalization response and can serve as a basis for similar research in other flowering bulbs.

### Data availability

Sequence data are available in the ArrayExpress database (www.ebi.ac.uk/arrayexpress) under accession number E-MTAB-2825. Parameters used for bioinformatics analyses are provided as Supplementary Information.

## Additional files

Additional file 1: Figure S1. Validation of RNAseq gene expression by quantitative polymerase chain reaction (qPCR). Additional file 2: Figure S2. Visual representation of main GO slim. Additional file 3: Figure S3. Protein sequence alignments between selected candidate genes and their putative orthologues in other species. Additional file 4:
**Parameters used for bioinformatic analyses.** Supplemental information on performed bioinformatics analyses.
